# Identifying and addressing gaps in reproductive health education for adolescent girls with type 1 diabetes

**DOI:** 10.1371/journal.pone.0206102

**Published:** 2018-11-06

**Authors:** Jaden R. Kohn, Marisa E. Hilliard, Sarah K. Lyons, Karin A. Fox, Jake A. Kushner

**Affiliations:** 1 Baylor College of Medicine, Houston, Texas, United States of America; 2 Department of Pediatrics, Baylor College of Medicine and Texas Children’s Hospital, Houston, Texas, United States of America; 3 Department of Obstetrics and Gynaecology, Division of Maternal-Fetal Medicine, Baylor College of Medicine, Houston, Texas, United States of America; 4 McNair Medical Institute, Baylor College of Medicine, Houston, Texas, United States of America; University of Illinois at Chicago, UNITED STATES

## Abstract

**Aims:**

Adolescent girls with diabetes are at risk for adverse pregnancy outcomes due to age, risk-taking behavior, poor glycemic control, and lack of knowledge. Our aims were to assess attitudes and behaviors related to reproductive health education (RHE) among diabetes healthcare providers and adolescent girls with diabetes, and to pilot a brief clinic-based RHE intervention.

**Methods:**

We surveyed 29 providers and 50 adolescent girls with type 1 diabetes about RHE experiences, attitudes, and behaviors. We piloted the RHE intervention with 9 adolescent-parent dyads.

**Results:**

50% of providers were very uncomfortable discussing pregnancy or contraception. Most (72%) did not proactively initiate RHE; common barriers included insufficient time and subject knowledge. Fewer than 10% recommended long-acting reversible contraceptives. A minority (10%) of adolescents had discussed pregnancy or contraception with a provider. RHE sessions lasted a median of 16 (range 13–24) minutes, and there were promising trends for changes in adolescents’ self-efficacy and intentions to use contraception and seek preconception counseling and in their knowledge of reproductive health.

**Conclusion:**

Adolescent girls with diabetes rarely receive education on pregnancy and contraception due to provider discomfort, limited knowledge, and limited time. RHE using easily-accessible materials with an educator may help address this gap in care.

## Introduction

### Risks of unintended pregnancy for adolescent girls with diabetes

Adolescent pregnancy is common and most pregnancies are unintended.[1, 2] In 2011 in the United States, 5.2% of girls age 15–19 years became pregnant and 3.1% gave birth;[3] in 2016, there were more than 200,000 births to adolescent mothers.[4] Adolescent pregnancy is a risk factor for reduced educational attainment and higher rates of unemployment, incarceration, and next-generation adolescent pregnancy.[5] Additionally, adolescent childbearing accounts for nearly $9.4 billion in annual U.S. federal spending due to the costs of public healthcare, welfare, and incarceration.[5]

Adolescent girls with diabetes are at risk for unintended pregnancy due to lack of knowledge, risky behavior, and irregular menses due to impaired glycemic control. Adolescent girls with diabetes often believe themselves to be at low risk of pregnancy and associated complications and are unaware of the need to use effective contraception.[6, 7] Adolescents with diabetes also exhibit risky behaviors (including unprotected sexual intercourse) more frequently than their peers without diabetes.[6, 8] Glycemic control worsens during adolescence for physiologic and psychologic reasons. Physiologically, hormonal changes lead to fluctuating insulin resistance during the menstrual cycle, resulting in higher postprandial glucose levels and more frequent hyperglycemia during the luteal phase.[9–11] Psychologically, many adolescents with diabetes experience diabetes-specific conflict with caregivers and are at increased risk for depression and anxiety, which impair diabetes management and worsen glycemic control.[12–14] Poor glycemic control leads to greater variability in the menstrual cycle,[15, 16] which makes it more difficult for an adolescent to know when she is at risk of conceiving. Together, these factors result in elevated risk of unplanned pregnancy.

Pregnancy with elevated blood glucose levels and pregnancy in adolescence are both associated with serious maternal and fetal risks.[17, 18] Fetal anomalies are four to ten times more common in women with diabetes;[17, 19] these develop during the first seven weeks of pregnancy and glycemic control is the most modifiable risk factor.[20–23] During pregnancy, women with diabetes are at increased risk for diabetic complications (hypoglycemia, diabetic ketoacidosis, and accelerated retinopathy), as well as pregnancy complications (miscarriage, preeclampsia, fetal growth restriction, macrosomia, stillbirth, preterm birth, and cesarean delivery).[17, 24] Compounding these risks, adolescent pregnancy is associated with higher rates of preterm delivery and low birth weight.[18, 25]

### Importance of reproductive health education related to diabetes

Preconception care for women with diabetes reduces the risk of major congenital anomalies and improves pregnancy outcomes;[26, 27] the American Diabetes Association (ADA) recommends that “starting at puberty, preconception counseling should be incorporated into routine diabetes care for all girls of childbearing potential” and “family planning should be discussed and effective contraception should be prescribed and used until a woman is prepared and ready to become pregnant.”[28] However, whether these guidelines are implemented into clinical practice for adolescents with diabetes is unknown.

To facilitate reproductive health education (RHE), Charron-Prochownik and colleagues developed a curriculum for adolescent girls with diabetes–Reproductive-health Education and Awareness of Diabetes in Youth for Girls (READY-Girls). It discusses the relationships between diabetes and puberty, pregnancy, contraception, and preconception counseling, and has been shown to improve knowledge as well as intentions to use birth control and to plan a pregnancy.[29–31] The ADA publishes an RHE booklet (Diabetes and Reproductive Health for Girls), which is adapted from the READY-Girls curriculum and available for free.[32] We could find no data in the literature regarding implementation of the “Diabetes and Reproductive Health for Girls” booklet in pediatric diabetes practices in the United States. We sought to identify gaps in RHE in our pediatric endocrinology clinic and to evaluate the feasibility of a brief interactive educational intervention using the ADA “Diabetes and Reproductive Health for Girls” booklet for RHE with an educator.

### Study objectives

The objectives of this study were to assess the attitudes and behaviors regarding RHE in diabetes healthcare professionals and in adolescent girls with diabetes, and to pilot the delivery of the ADA’s “Diabetes and Reproductive Health for Girls” booklet in a clinical setting with a health educator. We hypothesized that adolescents would report infrequent RHE by diabetes providers, that providers would report substantial barriers to providing RHE, and that delivering a brief clinic-based RHE intervention based on the ADA booklet would improve participant’s attitudes and knowledge related to RHE.

## Materials and methods

### Study design

We conducted two studies at a large pediatric endocrinology clinic in an academic medical center with a monthly patient volume of 240±36 visits for type 1 diabetes. All parts of this study were approved by the Baylor College of Medicine Institutional Review Board prior to any data collection (H-36074, H-34821). Written informed consent was obtained from the adolescent participants’ parent or legal guardian, and assent was obtained from the adolescent participant. First, to understand baseline perceptions about RHE and to assess attitudes and behaviors related to RHE, we conducted cross-sectional surveys of two populations: 1) diabetes healthcare providers (DHPs) and 2) adolescent girls with diabetes. Second, we conducted a small pilot study of the ADA’s RHE booklet (Diabetes and Reproductive Health for Girls, adapted from the validated READY-Girls curriculum) in a session with a trained-health educator, adolescent, and her parent. We evaluated patient and parent knowledge, attitudes, and beliefs about reproductive health through pre- and post-surveys. We also assessed the acceptability of the booklet and the discussion.

### Cross-sectional surveys

We administered two surveys: one to the pediatric endocrinology medical staff and one to female adolescent patients. No compensation was provided for participation in either survey.

#### Provider survey

For the provider survey, all physicians, nurse practitioners, and diabetes educators in the pediatric endocrinology clinic (n = 45) were eligible to participate. The survey was administered on paper via staff mailboxes. Participation was voluntary, and responses were anonymous. Completed surveys were returned in a sealed envelope to a secure collection box.

The survey contained 13 questions assessing provider characteristics (i.e., degree, proportion of regular patient panel with diabetes), attitudes about RHE (i.e., perceived importance of RHE during a diabetes care visit, comfort in providing RHE on various topics, factors that prompt delivery of RHE, barriers that hinder delivery of RHE, who should assume primary responsibility for providing RHE to female adolescent patients with diabetes), and RHE-related care behaviors (i.e., perceived frequency of delivering RHE to adolescent girls with diabetes, whether RHE discussions occurred with a parent in and/or out of the room, frequency of referrals to a gynecologist and for what indications).

#### Patient survey

For the patient survey, eligibility criteria were: (a) diagnosis of type 1 diabetes, according to ADA criteria,[33] for at least 6 months; (b) age 12–18 years; (c) female; (d) fluent in English. Data were collected for four months. Registration staff in clinic identified patients meeting these criteria upon arrival for their appointment and invited eligible patients to participate in the survey using a standardized script. The one-page paper survey contained a cover page with informed consent language, signed by the parent; the survey was then completed by the adolescent in the waiting room. Responses were anonymous, and no electronic health data was collected. Completed surveys were returned in a sealed envelope to a medical assistant.

The survey was designed for this study and contained 11 questions assessing demographic and clinical characteristics (i.e., age, race/ethnicity, duration of diabetes), previous exposure to RHE (including whether the adolescent had ever discussed puberty, pregnancy, birth control, and preconception counseling with a healthcare professional and at what age), and reproductive health behaviors (i.e., use of contraception, history of sexual activity, history of pregnancy). Respondents also indicated whether a parent had reviewed their responses. Lastly, respondents indicated whether they had participated in the RHE session with the “Diabetes and Reproductive Health for Girls” booklet; if so, these respondents were excluded from analysis.

### Pilot study of ADA RHE booklet

We conducted a pilot study to assess the feasibility of implementing a session to teach the basics of reproductive health in a clinical setting with a health educator. Patient eligibility criteria included: (a) diagnosis of type 1 diabetes, according to ADA criteria, for at least 6 months; (b) age 12–18 years; (c) female; (d) fluent in English; and (e) no current participation in another diabetes research study. Eligible patients were identified from the clinical schedule.

To recruit patients for the study, families received a letter by mail two to three weeks prior to the clinic appointment as well as a phone call one week prior to the appointment. If the family agreed to participate, they were asked to arrive fifteen minutes prior to their appointment to complete the informed consent process. One parent and the adolescent then each completed pre-intervention surveys; participants were instructed to complete questionnaires independently. After the adolescent was seen by her clinician, the adolescent and her parent/guardian participated in an RHE session with a trained health educator; the session was held in a private room in the clinic. The “Diabetes and Reproductive Health for Girls” booklet was used as a visual and informational aid explained by the educator. The adolescent and her parent were invited to ask questions freely during the session, and at the end, the adolescent was invited to ask questions privately, with the parent outside the room. Afterwards, the adolescent and parent completed post-intervention surveys. The time required for the educational session was measured, excluding the time required to complete the pre- and post-surveys. Families received reimbursement for parking as compensation for participation.

The pre- and post-intervention surveys were administered to patients and parents to assess knowledge, attitudes, intentions, and behaviors related to reproductive health. To assess the educational session with a health educator and the “Diabetes and Reproductive Health for Girls” booklet, we used the Reproductive Health Attitudes and Behaviors (RHAB) instrument,[34] which had previously been used to evaluate the efficacy of the READY-Girls curriculum,[29] but modified the instrument for brevity. The revised RHAB contains 25 questions and assesses six domains using a five-point Likert scale: (1) self-efficacy to use contraception and seek preconception counseling; (2) intentions to use contraception and seek preconception counseling; (3) susceptibility to pregnancy and adverse pregnancy outcomes, (4) severity of unintended pregnancy and adverse pregnancy outcomes, (5) benefits to using contraception and seeking preconception counseling, and (6) barriers to using contraception and seeking preconception counseling. Items assessing previous experiences with RHE were also included.[29] We also administered a 23-item validated true/false scale, used in prior assessments of READY-Girls,[29] to measure change in knowledge of reproductive health and diabetes (Appendix 3).

In the pre-intervention survey, data on demographic characteristics including race/ethnicity, education status, and religious background were collected from parents and adolescents. Adolescents also reported on their current use of contraception, sexual activity, and pregnancy history. The post-intervention survey sought feedback about the educational session from the adolescent and the parent, regarding the length of the book and the duration of the discussion; whether there were parts of the book and discussion that the participant did and did not like; whether anything made the participant feel uncomfortable, embarrassed or upset; and if there was anything else that could improve the educational session. Clinical data including adolescent age, duration of diabetes, menarcheal status, and hemoglobin A1c (HbA1c) were extracted from the electronic health record.

### Statistical analysis

For analysis of the baseline surveys and pilot study, descriptive statistics were generated. Normality of data was assessed using the Shapiro-Wilk tests before calculating measures of central tendency; Median and range are presented for data without a normal distribution, and mean and standard deviation are presented for data. Wilcoxon matched-pair signed-rank tests were conducted to compare Likert responses on pre- and post-intervention surveys. Analyses were performed using STATA/IC 14.2 for Mac (StataCorp, College Station, TX) and used a significance level of α = 0.05.

## Results

### Cross-sectional survey: Providers

Provider attitudes toward RHE are shown in Table 1. Of 45 providers who were eligible, 29 (64%) responded, including 22 pediatric endocrinologists, four nurse practitioners, and three diabetes educators.

**Table 1:** Diabetes care provider attitudes and behaviors regarding RHE (n = 29).

**Importance of RHE**	**Less important**	**Equally important**	**More important**
Compared to other clinical duties	21%	72%	7%
**Frequency of RHE discussion**	**Never**	**One or few visits**	**Most visits**
Puberty	7%	54%	39%
Pregnancy	29%	60%	11%
Contraception	39%	54%	7%
Preconception counseling	68%	32%	0%
**Comfort level with RHE**	**Very uncomfortable**	**Somewhat comfortable**	**Very comfortable**
Puberty	0%	21%	79%
Pregnancy	21%	62%	17%
Contraception	45%	48%	7%
Preconception counseling	55%	38%	7%
**Privacy when providing RHE**	**Parent in room**	**Parent out of room**	**With and without parent**
Parent present or absent	21%	10%	69%
**Responsibility for providing RHE**	**Diabetes care provider**	**Obstetrician-Gynecologist**	**PCP / Adolescent Med**
Puberty	94%	0%	6%
Pregnancy	51%	40%	9%
Contraception	14%	64%	22%
Preconception counseling	15%	73%	12%

Respondents could indicate >1 provider for receipt of RHE, thus responses sum to more than 100%.

Nearly 80% of providers believed that RHE was equally important or more important compared to other clinical responsibilities. Most providers reported counseling patients on puberty at one visit (18%), a few visits (36%), or most visits (39%). Nearly 30% reported never discussing pregnancy and nearly 40% reported never discussing contraception with their patients. All providers reported being somewhat or very comfortable discussing puberty. However, 21% of providers were very uncomfortable discussing pregnancy. Additionally, 50% of providers reported being very uncomfortable when discussing contraception and preconception counseling.

Providers endorsed multiple reasons for initiating RHE, including in response to an adolescent or parent question (76%), learning the patient was sexually active (76%), the development of a reproductive health complication related to diabetes such as irregular menses or candida vulvovaginitis (52%), or at menarche (38%). Age (55%) and Tanner stage (38%) were also commonly reported triggers for RHE, with a broad range of ages (10–16 years) and Tanner stages (2–5) endorsed.

Most providers reported that RHE occurred both with and without a parent in the room (69%); however, 21% led discussions always with the parent present and 10% always with the parent absent.

Reported barriers to providing RHE included insufficient time (52%) and lack of subject matter knowledge (31%). Other barriers reported included prioritization of diabetes control (10%), maturity level of the patient (3%), and lack of training for the discussion (3%).

Twenty-two providers (76%) reported providing referrals for reproductive healthcare in the past year. Of these, referral specialties included pediatric gynecology (82%), adolescent medicine (14%), or adult gynecology (4%), for indications including irregular menses, vaginal discharge, sexually transmitted infection, routine pelvic exam, contraception, and pregnancy.

When counseling about contraception, most providers would recommend oral contraceptive pills (69%) or male condoms (45%). Shorter-acting hormonal contraceptives were infrequently recommended: patch 17%, depot medroxyprogesterone acetate injection 10%, and vaginal ring 10%. Long-acting hormonal methods were rarely recommended; only 10% of providers had recommended an intrauterine device (IUD) and 7% had recommended a subdermal arm implant.

### Cross-sectional survey: Adolescents

We collected 50 responses from adolescents; respondent characteristics appear in Table 2. Adolescents in our sample were a mean of 14.6 ± 1.9 years in age. The duration of diabetes was a median of 3 years and ranged from 6 months to 11 years. Regarding race/ethnicity, 46% of participants identified as Hispanic, 30% non-Hispanic white, and 24% non-Hispanic black. Six adolescents reported sexual activity, and that their reported sexual debut occurred between ages 15 and 18 years. Of these six, two used long-acting reversible contraception (one IUD and one subdermal arm implant), two used male condoms, and two used the withdrawal method. One sexually active patient reported a prior pregnancy. Six reported use of short-acting hormonal contraceptives but no sexual activity, suggesting that contraceptive use was for medical indications.

**Table 2:** Adolescent characteristics. Shown as N, % or median (range) or mean± SD (range).

Cross-sectional survey (n = 50)		Pilot study (n = 9)
**Demographics**		
Age (years)	14.6 ± 1.9 (12–18)	16.6 ± 2.1 (12.9–18.7)
Diabetes duration (years)	3 (0.5–11)	2.7 (0.5–15.8)
HbA1c at visit	-	8.3 ± 2.3 (5.3–12.9)
Ethnicity		
Hispanic	23, 46%	1, 11%
Non-Hispanic White	15, 30%	3, 33%
Non-Hispanic Black	12, 24%	4, 44%
Asian	0, 0%	1, 11%
Religion		
Protestant or Catholic	-	5, 55%
None	-	4, 44%
Maternal education		
High school diploma or less	-	2, 22%
Some college or technical school	-	3, 33%
Bachelor’s degree or more	-	4, 44%
Annual household income ($)		
< 20,000	-	3, 33%
20,000–60,000	-	2, 22%
60,000–100,000	-	2, 22%
> 100,000	-	2, 22%
**Sexually active**		
No	38, 76%	6, 67%
Blank	06, 12%	1, 11%
Yes	06, 12%	2, 22%
Age at coitarche (years)	15 (n = 2), 16 (n = 3), 18 (n = 1)	15, 16
**History of pregnancy**		
No	45, 90%	9, 100%
Blank	04, 8%	0, 0%
Yes	01, 2%	0, 0%
**Contraceptive use**		
No	38, 76%	8, 89%
Yes	12, 24%	1, 11%
*Non-hormonal methods*		
Withdrawal	2, 4%	1, 11%
Male condom	2, 4%	1, 11%
Diaphragm	-	1, 11%
*Short-acting hormonal methods*		
Oral contraceptive pill	4, 8%	0, 0%
Transdermal patch	1, 2%	0, 0%
DMPA injection	1, 2%	0, 0%
*Long-acting hormonal methods*		
Intrauterine device	1, 2%	0, 0%
Subdermal arm implant	1, 2%	0, 0%
**Parent reviewed survey responses**		
No	41, 84%	9, 100%
Yes	8, 16%	0, 0%

Adolescent experiences with RHE are in Table 3. More than 60% reported that a healthcare professional had discussed puberty with her; nearly half reported that a discussion had occurred with a diabetes physician or nurse practitioner. Discussions about puberty were initiated at a mean of 12.5 ± 1.9 years with a diabetes physicians or nurse practitioner and at a mean of 13.0 ± 2.0 years with a diabetes educator. However, 78% of adolescents reported that they had never discussed pregnancy, 71% had never discussed contraception, and 84% had never discussed preconception counseling with any healthcare professional. Few adolescents reported ever discussing pregnancy (10%), contraception (14%), or preconception counseling (10%) with her diabetes physician or nurse practitioner.

**Table 3:** Previous experiences with RHE. Shown as N, % or mean± SD (range).

	Cross-sectional survey, adolescent (n = 50)		Pilot study, adolescent (n = 9)		Pilot study, parent (n = 9)
Topic	N, %	Age (years)	N, %	Age (years)	N, %
**Puberty**					
Never discussed	19, 39%	-	6, 67%	-	3, 33%
Discussed	30, 61%	-	3, 33%	-	6, 67%
Diabetes doctor or NP	23, 47%	12.5 ± 1.9 (8–18)	3, 33%	15, 16, 16	2, 22%
Diabetes educator	12, 24%	13.0 ± 2.0 (8–15)	2, 22%	15, 16	2, 22%
Primary care physician	8, 016%	12.6 ± 2.4 (8–16)	0, 0%	-	3, 33%
Obstetrician-gynecologist	5, 010%	13.9 ± 1.3 (12–15)	0, 0%	-	1, 11%
*Primary information source*	-		Parent 56%, TV/movies 44%, Internet 44%, School 33%		School 78%, Parent 67%, Internet 44%
**Pregnancy**					
Never discussed	38, 78%	-	8, 89%	-	3, 33%
Discussed	11, 22%	-	1, 11%	-	6, 67%
Diabetes doctor or NP	5, 10%	14.2 ± 2.9 (11–18)	1, 11%	16	2, 22%
Diabetes educator	4, 8%	12.8 ± 1.0 (12–14)	0, 0%	-	2, 22%
Primary care physician	1, 2%	16 (16)	0, 0%	-	3, 33%
Obstetrician-gynecologist	1, 2%	18 (18)	0, 0%	-	1, 11%
*Primary information source*	-		Parent 56%, TV/movies 44%, Internet 44%, School 33%		School 78%, Parent 67%, Internet 44%
**Contraception**					
Never discussed	35, 71%	-	7, 78%	-	5, 56%
Discussed	14, 29%	-	2, 22%	-	4, 44%
Diabetes doctor or NP	7, 14%	14.5 ± 2.4 (11–18)	0, 0%	-	1, 11%
Diabetes educator	3, 6%	13.3 ± 0.6 (13–14)	0, 0%	-	1, 11%
Primary care physician	1, 2%	16 (16)	0, 0%	-	2, 22%
Obstetrician-gynecologist	5, 10%	15.0 ± 0.7 (14–16)	2, 22%	-	1, 11%
*Primary information source*	-		Parent 33%, Internet 33%, School 33%, Friend 22%		School 67%, Parent 56%, Internet 22%
**Preconception counseling**					
Never discussed	41, 84%	-	8, 89%	-	9, 100%
Discussed	8, 016%	-	1, 11%	-	0, 0%
Diabetes doctor or NP	5, 10%	15.2 ± 2.8 (11–18)	0, 0%	-	0, 0%
Diabetes educator	4, 8%	13.3 ± 2.6 (11–17)	0, 0%	-	0, 0%
Primary care physician	0, 0%	-	0, 0%	-	0, 0%
Obstetrician-gynecologist	1, 2%	18 (18)	1, 11%	-	0, 0%
*Primary information source*	-		Friend 11%, Internet 11%		-

If an adolescent indicated that she had discussed the RHE topic, she could indicate all applicable health care professional roles–thus responses for each category sum to more than 100%.

### Pilot study of the ADA’s RHE materials

To pilot test the implementation of a RHE session using the “Diabetes and Reproductive Health for Girls” booklet with a health educator in our clinic, we contacted 71 families over four months. 61 families were unable to be successfully contacted by phone or declined to participate (typically citing lack of time or interest). One family completed the RHE intervention session but did not complete the post-intervention survey. Nine patient-parent dyads completed the full session and both pre- and post-surveys.

Adolescent characteristics are shown in Table 2. Participants were a mean of 16.6 ± 2.1 years of age. The duration of diabetes was a median of 2.7 years (range 0.4 to 15.8 years); mean HbA1c at the visit was 8.3±2.3% and ranged from 5.3 to 12.9%. One participant identified as Hispanic, three as non-Hispanic white, four as non-Hispanic black, and one as Asian. More than half (55%) had a household income of less than $60,000 per year; the same proportion had a mother who had attained less than a college education. One participant was sexually active and reported use of barrier contraceptive methods (including male condoms, a diaphragm, and withdrawal). No participants reported use of short- or long-acting hormonal contraception.

Participants’ previous experiences with RHE are shown in Table 3. Three participants indicated discussing puberty with a diabetes provider, which occurred at 15 or 16 years of age. One participant reported discussing pregnancy with a diabetes provider at age 16. Nearly 80% reported never discussing contraception with any healthcare provider; of those who had discussed contraception, these discussions had occurred only with an obstetrician-gynecologist. The primary sources of information about puberty, pregnancy, and contraception were parents, internet, television & movie media, and school classes.

All parental survey respondents were mothers. The majority (78%) reported that it was important to discuss reproductive health during a diabetes care visit. However, mothers perceived that RHE with a diabetes provider had been minimal (Table 2): two mothers believed their daughters had been given information about puberty and pregnancy, one believed her daughter had received information about contraception, and no mothers had heard of preconception counseling. Five mothers believed their daughter had never discussed contraception with any healthcare provider. Two mothers preferred to be present for all RHE discussions, four mothers preferred to be in and out of the room, and three mothers had no preference.

Delivery of RHE with a trained health educator using the “Diabetes and Reproductive Health for Girls” booklet required a median of 16 minutes, ranging from 13 to 24 minutes. Table 4 reports pre-and post-intervention scores on the modified RHAB instrument. For adolescents, we found a notable increase in intentions, self-efficacy, and knowledge, though the sample size is small and the long-term impact on behaviors remains unknown. When comparing maternal attitudes before and after the intervention, we found a notable increase in the perceived benefits of preconception counseling and contraceptive use.

**Table 4:** Change on Reproductive Health Attitudes and Behaviors (RHAB) domains, before and after the educational session with the “Diabetes and Reproductive Health for Girls” booklet from the American diabetes association.

**Adolescents (n = 9)**	**Scale**	**Pre**	**Post**	***p-*value**
Perceived benefits	3–15	12 (11–13)	14 (12–14)	0.23
Perceived barriers	2–10	20 (2–3)	20 (2–6)	0.22
Perceived susceptibility	3–15	11 (8–11)	90 (8–11)	0.55
Perceived severity	3–15	14 (13–15)	13 (12–15)	0.20
Intentions	2–10	80 (5–10)	10 (10–10)	0.02
Self-efficacy	6–30	21 (13–28)	29 (25–30)	0.01
Knowledge	0–21	15 (13–16)	19 (16–20)	0.01
**Mothers (n = 9)**	**Scale**	**Pre**	**Post**	***p-*value**
Perceived benefits	1–50	40 (3–5)	50 (5–5)	0.03
Perceived barriers	1–50	10 (1–3)	20 (1–3)	0.61
Perceived susceptibility	3–15	10 (6–10)	11 (7–13)	0.28
Perceived severity	3–15	14 (11–15)	15 (11–15)	1.00

Shown as median (interquartile range).

Feedback from adolescents about the RHE session with the “Diabetes and Reproductive Health for Girls” booklet included: “I got to ask questions and it wasn't uncomfortable”, “I asked plenty of questions, and the book provided plenty of details and things I did not know about”, and “it told me a lot of things I never knew about sex and baby development and how many ways you could prevent a baby to not have any problems.” Adolescents also said, “It made me a little scared because when I do start having sex, I don’t want a baby or have anything bad happen to it” and “I have never spoken to my parents about sex so it was awkward at first, but then it got better.” Feedback from mothers included: “I really got a lot out of the discussion. You can have a normal baby with diabetes”, “I appreciate it because it made my follow-up as a parent easier”, “Visual artwork and discussion very clear”, “Provided good overview of information”, and “It was very good and patients (girls) should do this.” One parent noted that “it was very scary” and expressed concerns about discussing contraception. No participants believed that the length of the book or length of the discussion were too long or too short. Five participants indicated they would also like to have materials available to take home with them for later review after the RHE session.

## Discussion

We present novel findings regarding gaps within RHE from the perspectives of diabetes providers and a diverse sample of adolescent girls with diabetes. We also discuss a small pilot study to assess the delivery of RHE materials in a busy clinical setting. Our study sample included a larger proportion of younger, non-White, and low-income participants compared to previous studies about RHE for adolescent girls with diabetes, and therefore provides insight into reproductive health issues in a broader population.[29, 31] Importantly, we found that most surveyed healthcare providers indicated that they preferred to begin discussions at older ages, which may be too late to provide anticipatory guidance, especially for a population at-risk for complications of unintended pregnancy. Notably, although most providers believed that they discuss pregnancy and contraception, very few adolescent respondents recalled these conversations–suggesting that RHE may need to be both more frequent and consistent.

A previous study on sexual behaviors demonstrated a 40% rate of sexual activity in adolescents with diabetes and a 60% rate in adolescents without diabetes;[6] 12% of adolescents in our cross-sectional study and 22% in our pilot study reported sexual activity, though actual rates may be higher as some did not respond to the question and 16% indicated in the cross-sectional survey that a parent had reviewed their answers (potentially biasing the responses). Most adolescents who reported sexual activity used the withdrawal method or condoms, consistent with previous literature suggesting that half of sexually active adolescent girls with diabetes were not using highly effective birth control.[35] Only 25% of the adolescents in our study used long-acting reversible contraceptive methods (LARCs), such as an IUD or subdermal implant.[36–38] Notably, few providers indicated that they would recommend a LARC, despite evidence that LARCs are the safest, most effective, and most successful contraceptive method for adolescents with diabetes.[36–38]

Additionally, provider discomfort, lack of subject knowledge, and insufficient time were reported to hinder the frequency and consistency of RHE–highlighting the need for effective, efficient tools and training for provider and/or the need for close partnership with (and easy patient access to) a provider specializing in reproductive health, in order to improve the delivery of reproductive health information. This partnership may be essential, as a high proportion of diabetes healthcare professionals indicated a belief that an obstetrician-gynecologist should be responsible for counseling adolescent girls with diabetes about pregnancy, contraception, and preconception counseling. To be effective, RHE must be delivered by providers with sufficient knowledge, comfort, and time to discuss pregnancy, contraception, and preconception counseling, in addition to discussing puberty. Lastly, we found that media (television, movies, and the internet) was a very common source of information on reproductive health topics for this population, underscoring how important it is to ensure that accurate information is available from these sources for adolescent girls.

Our implementation pilot indicates that a single, approximately 15-minute session led by a health educator using highly-accessible materials is able to be delivered in a real clinic setting, resulting in improvements in knowledge, intentions, and self-efficacy. Given the substantial barriers discussing reproductive health, it may be helpful for practices to identify a designated health educator to deliver RHE or to consider group education sessions using the available material for RHE. Consistent, standardized initiation of RHE in early adolescence with a health educator may help to normalize discussions about reproductive health. Our survey data demonstrate that a critical gap exists in the current delivery of care for adolescent girls with diabetes; among the families who agreed to participate in our RHE pilot, our findings suggest that using the easily-accessible ADA educational materials with an educator has the potential to improve knowledge and some attitudes related to reproductive health. However, the low consent rate for the RHE session may raise questions about whether an in-person educational session is a broadly-acceptable approach to RHE. Lastly, while the material in this study did not contain information about LARC methods, we recommend discussions about contraception include safety and effectiveness of LARCs in adolescents with diabetes.

As with all research, the limitations of this study must be considered. First, our study was conducted at a single institution in the state of Texas, which may limit generalizability of findings to other settings with different demographics and policies regarding RHE in schools. However, the ethnic diversity of the sample is a strength for external validity, given increasing rates of type 1 diabetes in Hispanic youth.[39] Unfortunately, outcome assessments were not available in Spanish, limiting our sample to those fluent in English. Additionally, the pilot study was small; it may be that parents who consented for their child to participate perceive RHE as more important, are more comfortable with RHE discussions, or perceive that their daughters received less RHE previously–this may not apply to all parents of adolescent girls with diabetes. Indeed, as a high proportion declined participation, alternate strategies to introduce RHE may be needed to reduce discomfort with the topic and/or the burden of staying after a clinic visit for additional education. As such, this work will need to be replicated in a larger, more representative sample to determine generalizability and broad acceptability. Furthermore, as our surveys inquired about attitudes, social desirability bias may have influenced respondents to provide answers that would be perceived well (i.e. DHPs want to provide comprehensive care, adolescents provide answers acceptable to parents who may review survey responses). Additionally, we did not track the number of patients eligible for the cross-sectional survey, limiting our ability to generate a response rate or to gather data on the population who declined to participate. Lastly, while our pilot intervention benefits from use of the “Diabetes and Reproductive Health for Girls” booklet (which is adapted from the well-established READY-Girls curriculum), it is not equivalent to the READY-Girls intervention and requires ongoing research in a larger sample size to determine its utility and impact. Despite limitations, our study adds useful knowledge to existing literature regarding the gaps in the current delivery of RHE, the attitudes of providers, adolescents with diabetes, and parents towards RHE, and the potential to address gaps using the ADA’s easily-accessible “Diabetes and Reproductive Health for Girls” booklet.

## Conclusion

Adolescent patients and their families should be educated early about the relationship between female reproductive health and diabetes. Diabetes healthcare professionals believe that RHE is equally as important as other clinical responsibilities and are comfortable counseling adolescents with diabetes about puberty; however, they often fail to adequately discuss pregnancy and contraception due to provider discomfort, limited knowledge, and limited time. Providing standardized RHE in early adolescence for all female patients with diabetes as part of routine clinical care, using easily-accessible materials based on validated research, can address this critical gap.

## Supporting information

S1 File Surveys used in the study. Included are the surveys utilized for the cross-sectional study of adolescents and health care providers, as well as the pre- and post-intervention surveys for the READY-Girls RHE study.
(ZIP)

S1 Dataset Raw data from the study. Files containing raw data analyzed in the production of this manuscript.
(ZIP)
