# Supplementary material for: Identifying and addressing gaps in reproductive health education for adolescent girls with type 1 diabetes
Source: PLoS One. 2018 Nov 6;13(11):e0206102. doi: 10.1371/journal.pone.0206102 (PMC6219771; doi:10.1371/journal.pone.0206102)
Supplement: S1 File — Included are the surveys utilized for the cross-sectional study of adolescents and health care providers, as well as the pre- and post-intervention surveys for the READY-Girls RHE study. (ZIP) [file pone.0206102.s001.zip › PLoS survey attach/Parent, pre-questionnaire_PLoS.docx]

**We are interested in learning more about your attitudes related to diabetes, puberty, and pregnancy, so that we can provide better care for your adolescent and other girls with diabetes. Please answer these questions honestly. The doctor/nurse and your adolescent will not see your answers to any question. There are no right or wrong answers. Please ask if you have a question on any item.**

*We would like to know a few things about you and your family.*

1. What is your relationship to your adolescent?

☐ Mother ☐ Father ☐ Step-parent ☐ Grandparent ☐ Legal guardian

1. Does your adolescent live with you full-time?

☐ Yes; go to question 3 ☐ No; please briefly describe the living arrangements in the space below

1. What is your race/ethnicity?

☐ Caucasian (non-Hispanic) ☐ Hispanic / Latino ☐ Black or African-American (non-Hispanic)

☐ Asian/Pacific Islander ☐ Other:

1. Is your adolescent in school full-time?

☐ yes ☐ no

1. What is your religion?

☐ None ☐ Catholic ☐ Protestant ☐ Orthodox ☐ Muslim

☐ Jewish ☐ Hindu ☐ Buddhist ☐ Other:

1. How important is religion in your life?

☐ Not important ☐ Important ☐ Very important

1. Do you identify with the same religion as your adolescent?

☐ Yes ☐ No

1. What is the highest degree or level of school you have completed?

☐ Up to 8^th^ grade ☐ Some high school, no diploma ☐ GED ☐ High school diploma

☐ Trade/technical training ☐ Some college, no degree ☐ Associate’s degree ☐ Bachelor’s degree

☐ Master’s degree ☐ Doctorate or professional degree

1. What was your total household income in the past year?

☐ Less than $20,000 ☐ $20,000-39,999 ☐ $40,000-$59,999 ☐ $60,000-79,999

☐ $80,000-99,999 ☐ $100,000-149,999 ☐ More than $150,000

*We would like to know more about discussions of puberty, sex, pregnancy, and birth control in the past.*

1. Has your adolescent ever received information about puberty and/or pregnancy from the following sources?

You ☐ Yes ☐ No

Brother or sister ☐ Yes ☐ No

Other relative ☐ Yes ☐ No

Friend ☐ Yes ☐ No

Boyfriend / sexual partner ☐ Yes ☐ No

School sex education class ☐ Yes ☐ No

TV/movies ☐ Yes ☐ No

Books/magazines ☐ Yes ☐ No

Internet ☐ Yes ☐ No

Primary/regular doctor ☐ Yes ☐ No

Diabetes doctor or nurse ☐ Yes ☐ No

Diabetes educator ☐ Yes ☐ No

Obstetrician-gynecologist ☐ Yes ☐ No

***Please circle the one source from which you believe she has received the most information.***

1. Has anyone ever told your adolescent that she should use some type of birth control when preventing a pregnancy?

You ☐ Yes ☐ No

Brother or sister ☐ Yes ☐ No

Other relative ☐ Yes ☐ No

Friend ☐ Yes ☐ No

Boyfriend / sexual partner ☐ Yes ☐ No

School sex education class ☐ Yes ☐ No

TV/movies ☐ Yes ☐ No

Books/magazines ☐ Yes ☐ No

Internet ☐ Yes ☐ No

Primary/regular doctor ☐ Yes ☐ No

Diabetes doctor or nurse ☐ Yes ☐ No

Diabetes educator ☐ Yes ☐ No

Obstetrician-gynecologist ☐ Yes ☐ No

***Please circle the one source from which you have received the most information.***

1. How comfortable are you talking with your adolescent about puberty?

☐ very uncomfortable ☐ uncomfortable ☐ neither comfortable nor uncomfortable ☐ comfortable ☐ very comfortable

1. How comfortable are you talking with your adolescent about sex?

☐ very uncomfortable ☐ uncomfortable ☐ neither comfortable nor uncomfortable ☐ comfortable ☐ very comfortable

1. How comfortable are you talking with your adolescent about pregnancy?

☐ very uncomfortable ☐ uncomfortable ☐ neither comfortable nor uncomfortable ☐ comfortable ☐ very comfortable

1. How comfortable are you talking with your adolescent about birth control?

☐ very uncomfortable ☐ uncomfortable ☐ neither comfortable nor uncomfortable ☐ comfortable ☐ very comfortable

1. What could your adolescent’s diabetes doctor/nurse/educator do to help you be more comfortable discussing these topics with your adolescent?

1. Do you think it is important to discuss puberty, sex, pregnancy & birth control during your adolescent’s diabetes visit?

☐ yes ☐ no

1. When discussing puberty, sex, pregnancy, and birth control, would you prefer to be in the room with your adolescent and the doctor/nurse/educator, or out of the room so that your adolescent can discuss this alone with the provider?

☐ I would like to be in the room with my adolescent for the entire discussion

☐ I would like to be out of the room for the entire discussion

☐ I would like to be in the room for part of the discussion, and out of the room for part of the discussion

☐ It does not matter to me

1. At what age do you think that your adolescent’s diabetes doctor/nurse/educator should begin to talk with you and your adolescent about the following things:
   - Puberty, menstrual cycles, and diabetes: _________
   - Pregnancy and the risks of high blood sugar for the mom & the fetus: ______
   - Pregnancy prevention (birth control): ______
2. When discussing puberty, sex, pregnancy, and birth control, would you prefer to talk with a female diabetes provider?

☐ Yes, I would prefer that we talk with a female provider ☐ No, the gender does not matter

*These questions ask about your beliefs about a possible pregnancy for your adolescent. In these questions, the word adolescent is used, but this does not assume that she will become pregnant as a teenager. These questions can also apply to any future pregnancy when your adolescent has become an adult woman.*

1. How much do you worry that your adolescent could become pregnant?

☐ not at all ☐ a little ☐ somewhat ☐ a moderate amount ☐ a lot

1. If your adolescent had an unplanned pregnancy, do you think that this problem would be

☐ not serious at all ☐ a little serious ☐ somewhat serious ☐ moderately serious ☐ very serious

1. If your adolescent developed health problems during a pregnancy, do you think that those problems would be

☐ not serious at all ☐ a little serious ☐ somewhat serious ☐ moderately serious ☐ very serious

1. How much do you worry that your adolescent could develop health problems during pregnancy?

☐ not at all ☐ a little ☐ somewhat ☐ a moderate amount ☐ a lot

1. If your adolescent’s baby developed health problems during a pregnancy, do you think that the problems would be

☐ not serious at all ☐ a little serious ☐ somewhat serious ☐ moderately serious ☐ very serious

1. How much do you worry that your adolescent’s baby could develop health problems during a pregnancy?

☐ not at all ☐ a little ☐ somewhat ☐ a moderate amount ☐ a lot

*These questions ask about your beliefs about pre-conception counseling. Pre-conception counseling is special medical care and advice that is given by a doctor, nurse, or educator before someone becomes pregnant / is planning a pregnancy.*

1. Has anyone ever told you that your adolescent should get preconception counseling?

☐ No ☐ Yes; if so, who? _____________________________________

1. Receiving preconception planning when planning a pregnancy would improve your adolescent’s chances of having a healthy baby:

☐ not at all ☐ a little ☐ somewhat ☐ a moderate amount ☐ a lot

1. How difficult would it be for your adolescent to follow the preconception counseling advice given by a health professional (e.g. keeping blood sugar in the normal range, taking more insulin injections, etc.)?

☐ no problem at all ☐ a little ☐ somewhat ☐ a moderate problem ☐ a big problem

1. Are there other things that your adolescent’s diabetes provider could do to help you and your adolescent feel more informed about puberty, pregnancy, and birth control related to diabetes?
