# Supplementary material for: Identifying and addressing gaps in reproductive health education for adolescent girls with type 1 diabetes
Source: PLoS One. 2018 Nov 6;13(11):e0206102. doi: 10.1371/journal.pone.0206102 (PMC6219771; doi:10.1371/journal.pone.0206102)
Supplement: S1 File — Included are the surveys utilized for the cross-sectional study of adolescents and health care providers, as well as the pre- and post-intervention surveys for the READY-Girls RHE study. (ZIP) [file pone.0206102.s001.zip › PLoS survey attach/Child, pre-questionnaire_PLoS.docx]

**We want to learn more about your attitudes related to diabetes, puberty, and pregnancy, so we can provide better health care for you and other girls with diabetes. Please answer the questions honestly. Your doctor and your parents will not see your answers. There are no right or wrong answers. Ask if you have a question on any item.**

*These questions ask about the information you have received in the past.*

1. Have you ever received information about puberty and/or pregnancy from any of the following sources?

Parent/step-parent ☐ Yes ☐ No

Brother or sister ☐ Yes ☐ No

Other relative ☐ Yes ☐ No

Friend ☐ Yes ☐ No

Boyfriend / sexual partner ☐ Yes ☐ No

School sex education class ☐ Yes ☐ No

TV/movies ☐ Yes ☐ No

Books/magazines ☐ Yes ☐ No

Internet ☐ Yes ☐ No

Primary/regular doctor ☐ Yes ☐ No

Diabetes doctor or nurse ☐ Yes ☐ No

Diabetes educator ☐ Yes ☐ No

Obstetrician-gynecologist ☐ Yes ☐ No

***Please circle the one source from which you have received the most information.***

1. Have you ever discussed with a diabetes healthcare provider (doctor, nurse) how diabetes affects puberty?

☐ no ☐ yes; please answer question 4

1. Have you ever discussed with a diabetes educator how diabetes affects puberty?

☐ no ☐ yes; please answer question 4

1. How old were you when diabetes & puberty was first discussed with a diabetes healthcare provider / educator? _______
2. Have you ever discussed with a diabetes healthcare provider (doctor, nurse) how diabetes affects pregnancy?

☐ no ☐ yes; please answer question 7

1. Have you ever discussed with a diabetes educator how diabetes affects pregnancy?

☐ no ☐ yes; please answer question 7

1. How old were you when diabetes & pregnancy was first discussed with a diabetes healthcare provider / educator? _____

*These questions ask about how comfortable you are talking about diabetes and puberty, sex, pregnancy, & birth control.*

1. How comfortable are you talking with a parent/guardian about these issues?

☐ very uncomfortable ☐ uncomfortable ☐ neither comfortable nor uncomfortable ☐ comfortable ☐ very comfortable

1. How comfortable are you talking with a diabetes doctor or nurse about these issues?

☐ very uncomfortable ☐ uncomfortable ☐ neither comfortable nor uncomfortable ☐ comfortable ☐ very comfortable

1. How comfortable are you talking with a diabetes educator about these issues?

☐ very uncomfortable ☐ uncomfortable ☐ neither comfortable nor uncomfortable ☐ comfortable ☐ very comfortable

1. How comfortable are you talking with an obstetrician-gynecologist about these issues?

☐ very uncomfortable ☐ uncomfortable ☐ neither comfortable nor uncomfortable ☐ comfortable ☐ very comfortable

1. With whom are you the most comfortable discussing diabetes and puberty, sex, pregnancy, & birth control?

☐ Parent ☐ Diabetes doctor or nurse ☐ Diabetes educator ☐ Obstetrician/gynecologist

☐ Someone else (please write who):

1. When discussing puberty, sex, pregnancy, and birth control, would you prefer to talk with a female doctor/nurse/educator?

☐ Yes, I would prefer to talk with a female ☐ No, the gender does not matter

1. When discussing puberty, sex, pregnancy, and birth control, would you prefer that your parents be in the room with you and the diabetes doctor/nurse/educator, or out of the room so that you are alone with the doctor/nurse/educator?

☐ I would like my parent(s) to be in the room for the entire discussion

☐ I would like my parent(s) to be out of the room for the entire discussion

☐ I would like my parent(s) to be in the room for part of the discussion, and out of the room for part of the discussion

☐ It does not matter to me

*These questions ask about your beliefs about pregnancy.*

1. How much do you worry that you could become pregnant?

☐ not at all ☐ a little ☐ somewhat ☐ a moderate amount ☐ a lot

1. If you had an unplanned pregnancy, do you think that this would be

☐ not serious at all ☐ a little serious ☐ somewhat serious ☐ moderately serious ☐ very serious

1. How much do you worry that you could develop health problems during pregnancy?

☐ not at all ☐ a little ☐ somewhat ☐ a moderate amount ☐ a lot

1. If you developed health problems during a pregnancy, do you think that those problems would be

☐ not serious at all ☐ a little serious ☐ somewhat serious ☐ moderately serious ☐ very serious

1. How much do you worry that your baby could develop health problems during your pregnancy?

☐ not at all ☐ a little ☐ somewhat ☐ a moderate amount ☐ a lot

1. If your baby developed health problems during a pregnancy, do you think that the problems would be

☐ not serious at all ☐ a little serious ☐ somewhat serious ☐ moderately serious ☐ very serious

1. Having normal blood sugar levels before becoming pregnant would improve your chances of having a healthy baby:

☐ not at all ☐ a little ☐ somewhat ☐ a moderate amount ☐ a lot

*These questions ask about your beliefs about pre-conception counseling. Pre-conception counseling is special medical care and advice that is given by a doctor, nurse, or educator before someone becomes pregnant / is planning a pregnancy.*

1. Before now, have any of the following ever told you that you should get pre-conception counseling?

Parent/step-parent ☐ Yes ☐ No

Brother or sister ☐ Yes ☐ No

Other relative ☐ Yes ☐ No

Friend ☐ Yes ☐ No

Boyfriend / sexual partner ☐ Yes ☐ No

School sex education class ☐ Yes ☐ No

Books/magazines ☐ Yes ☐ No

Internet ☐ Yes ☐ No

Primary/regular doctor ☐ Yes ☐ No

Diabetes doctor or nurse ☐ Yes ☐ No

Diabetes educator ☐ Yes ☐ No

Obstetrician-gynecologist ☐ Yes ☐ No

1. Receiving pre-conception planning when planning a pregnancy would improve your chances of having a healthy baby:

☐ not at all ☐ a little ☐ somewhat ☐ a moderate amount ☐ a lot

1. How difficult do you think it would be to seek pre-conception counseling when planning a pregnancy?

☐ no problem at all ☐ a little ☐ somewhat ☐ a moderate problem ☐ a big problem

1. How difficult do you think it would be to follow the pre-conception counseling advice given by a diabetes provider (keeping blood sugar in the normal range, taking more insulin injections, etc.)?

☐ no problem at all ☐ a little ☐ somewhat ☐ a moderate problem ☐ a big problem

1. When I am ready to plan a pregnancy, I intend to seek preconception counseling from a diabetes provider.

☐ definitely no ☐ probably no ☐ maybe ☐ probably yes ☐ definitely yes

*The next questions ask about your beliefs about birth control and pregnancy prevention.*

1. Has anyone ever told you that you should use some type of birth control when preventing a pregnancy?

Parent/step-parent ☐ Yes ☐ No

Brother or sister ☐ Yes ☐ No

Other relative ☐ Yes ☐ No

Friend ☐ Yes ☐ No

Boyfriend / sexual partner ☐ Yes ☐ No

School sex education class ☐ Yes ☐ No

TV/movies ☐ Yes ☐ No

Books/magazines ☐ Yes ☐ No

Internet ☐ Yes ☐ No

Primary/regular doctor ☐ Yes ☐ No

Diabetes doctor or nurse ☐ Yes ☐ No

Diabetes educator ☐ Yes ☐ No

Obstetrician-gynecologist ☐ Yes ☐ No

***Please circle the one source from which you have received the most information.***

1. Using birth control would prevent an unplanned pregnancy:

☐ not at all ☐ a little ☐ somewhat ☐ a moderate amount ☐ a lot

1. In the future, when I have sex, I intend to always use some type of birth control to prevent an unplanned pregnancy.

☐ definitely no ☐ probably no ☐ maybe ☐ probably yes ☐ definitely yes

1. Do you currently use a form of birth control (to prevent pregnancy or for other reasons)? Please check all that apply.

☐ No birth control; *please skip to question 36*

☐ Condom (male) ☐ Withdrawal (“pulling out”) ☐ Rhythm/calendar method

☐ Condom (female) ☐ Diaphragm ☐ Nuvaring ☐ Implantable device

☐ Birth control pills ☐ Injections/shots ☐ Intrauterine device (IUD)

1. How much of a problem for you is the cost of birth control? ☐ doesn’t apply

☐ no problem at all ☐ a little ☐ somewhat ☐ a moderate problem ☐ a big problem

1. How much of a problem for you is getting birth control? ☐ doesn’t apply

☐ no problem at all ☐ a little ☐ somewhat ☐ a moderate problem ☐ a big problem

1. What makes it difficult for you to get birth control?
2. How much of a problem for you is using birth control on a regular basis? ☐ doesn’t apply

☐ no problem at all ☐ a little ☐ somewhat ☐ a moderate problem ☐ a big problem

1. What makes it difficult for you to use birth control?

*These questions ask about your sexual activity. Remember that neither your doctor nor your parents will see your answers.*

1. Have you voluntarily had vaginal sex with someone of the opposite sex?

☐ no ☐ yes; If yes, how old were you when you first had voluntary vaginal sex?

1. Are you currently, or have you ever been, pregnant?

☐ no ☐ yes; If yes, how many times have you been pregnant?

*These questions ask about your confidence that you could do the thing named in each question.*

*“How confident am I that I could …”*

1. Get preconception counseling before I get pregnant?

☐ very unconfident ☐ unconfident ☐ maybe ☐ confident ☐ very confident

1. Change my insulin and diet to keep my blood sugar levels in normal range if I am planning a pregnancy (not yet pregnant).

☐ very unconfident ☐ unconfident ☐ maybe ☐ confident ☐ very confident

1. Wait on becoming pregnant until my blood sugar levels are within the normal range

☐ very unconfident ☐ unconfident ☐ maybe ☐ confident ☐ very confident

1. Convince my sexual partner that it is necessary to use birth control, even if he doesn’t want to use it.

☐ very unconfident ☐ unconfident ☐ maybe ☐ confident ☐ very confident

1. Use birth control each time I have sex when preventing a pregnancy.

☐ very unconfident ☐ unconfident ☐ maybe ☐ confident ☐ very confident

1. Delay sex if birth control is not available

☐ very unconfident ☐ unconfident ☐ maybe ☐ confident ☐ very confident

*Lastly, we want to know a few more things about you and your family.*

1. What is your race/ethnicity?

☐ Caucasian (non-Hispanic) ☐ Hispanic / Latino ☐ Black or African-American (non-Hispanic)

☐ Asian/Pacific Islander ☐ Other:

1. Are you in school full-time?

☐ yes ☐ no

1. Do you identify with a religion? If yes, please check the appropriate box.

☐ No religion ☐ Catholic ☐ Protestant ☐ Orthodox ☐ Muslim

☐ Jewish ☐ Hindu ☐ Buddhist ☐ Other:

1. How important is religion in your life?

☐ Not important ☐ Important ☐ Very important

**We are interested in learning about what you currently know about diabetes, puberty, pregnancy, and birth control. Make sure to read the questions carefully, and answer them as best you can. It is okay to get the answer wrong. Ask if you have a question on any item.**

☐ True ☐ False 1. Once a girl has begun having periods, she could become pregnant.

☐ True ☐ False 2. High blood sugar levels can cause irregular menstrual periods.

☐ True ☐ False 3. A girl/woman can become pregnant during her period.

☐ True ☐ False 4. A girl/woman cannot become pregnant the first time she has sexual intercourse.

☐ True ☐ False 5. To prevent pregnancy, birth control must be used each time a girl/woman has sex.

☐ True ☐ False 6. A girl/woman is most likely to become pregnant if she has sex about two weeks after her period.

☐ True ☐ False 7. A girl/woman with diabetes does not have an increased risk of vaginal infections.

☐ True ☐ False 8. A girl/woman with diabetes cannot have a healthy baby.

☐ True ☐ False 9. The target range for blood sugar during pregnancy in a girl/woman with diabetes is 100-200 mg/dl.

☐ True ☐ False 10. During pregnancy, the level of blood sugar in the fetus will be similar to the mother’s sugar.

☐ True ☐ False 11. High blood sugar levels during the first two months of pregnancy increase the risk of problems for the mom, but not the fetus.

☐ True ☐ False 12. If a girl/woman with diabetes has high blood sugar at conception (the moment they get pregnant), she has an increased risk of having a baby with birth defects.

☐ True ☐ False 13. A girl/woman with diabetes who has high blood sugar during pregnancy does not have an increased risk of miscarriage (sudden death of a fetus during pregnancy).

☐ True ☐ False 14. If a girl/woman with diabetes has high blood sugar during pregnancy, her baby could be a large baby, making delivery more difficult.

☐ True ☐ False 15. After a positive pregnancy test in a girl/woman with diabetes, the best way to prevent problems or harm to the fetus is getting blood sugar levels to the target range as soon as possible.

☐ True ☐ False 16. A girl/woman with diabetes has very few choices of birth control.

☐ True ☐ False 17. All birth control methods are less effective in girls/women with diabetes.

☐ True ☐ False 18. A girl/woman with diabetes cannot use any type of birth control pills.

☐ True ☐ False 19. A girl/woman with diabetes can use long-term birth control methods, such as an intrauterine device.

☐ True ☐ False 20. The sexual partner of a girl/woman with diabetes can use condoms.

☐ True ☐ False 21. Sex is like exercise and can cause low blood sugar (hypoglycemia) reactions.
