# Supplementary material for: Identifying and addressing gaps in reproductive health education for adolescent girls with type 1 diabetes
Source: PLoS One. 2018 Nov 6;13(11):e0206102. doi: 10.1371/journal.pone.0206102 (PMC6219771; doi:10.1371/journal.pone.0206102)
Supplement: S1 File — Included are the surveys utilized for the cross-sectional study of adolescents and health care providers, as well as the pre- and post-intervention surveys for the READY-Girls RHE study. (ZIP) [file pone.0206102.s001.zip › PLoS survey attach/Child, post-questionnaire_PLoS.docx]

**Now that you have talked with a diabetes provider about puberty, pregnancy, and birth control, we would like to know more about your attitudes and opinions, so we can provide better health care for you and other girls with diabetes. Please answer the questions honestly. Your doctor and your parents will not see your answers. There are no right or wrong answers. Ask if you have a question on any item.**

1. Program Name:
2. Which of the following methods of formal psychiatric training is provided to your residents?

- Didactic
- Elective clinical rotation
- Mandatory clinical rotation

☐ not at all

☐ a little

☐ somewhat

☐ a moderate amount ☐ a lot

1. How difficult do you think it would be to seek pre-conception counseling when planning a pregnancy?

☐ no problem at all ☐ a little ☐ somewhat ☐ a moderate problem ☐ a big problem

1. How difficult do you think it would be to follow the pre-conception counseling advice given by a diabetes provider (keeping blood sugar in the normal range, taking more insulin injections, etc.)?

☐ no problem at all ☐ a little ☐ somewhat ☐ a moderate problem ☐ a big problem

1. Which part of the preconception counseling would be the least difficult (easiest) to follow?

1. Which part of the preconception counseling would be the most difficult to follow?

1. When I am ready to plan a pregnancy, I intend to seek preconception counseling from a diabetes provider.

☐ definitely no ☐ probably no ☐ maybe ☐ probably yes ☐ definitely yes

*These questions ask about your beliefs about pregnancy.*

1. How much do you worry that you could become pregnant?

☐ not at all ☐ a little ☐ somewhat ☐ a moderate amount ☐ a lot

1. If you had an unplanned pregnancy, do you think that this would be

☐ not serious at all ☐ a little serious ☐ somewhat serious ☐ moderately serious ☐ very serious

1. How much do you worry that you could develop health problems during pregnancy?

☐ not at all ☐ a little ☐ somewhat ☐ a moderate amount ☐ a lot

1. If you developed health problems during a pregnancy, do you think that those problems would be

☐ not serious at all ☐ a little serious ☐ somewhat serious ☐ moderately serious ☐ very serious

1. How much do you worry that your baby could develop health problems during your pregnancy?

☐ not at all ☐ a little ☐ somewhat ☐ a moderate amount ☐ a lot

1. If your baby developed health problems during a pregnancy, do you think that the problems would be

☐ not serious at all ☐ a little serious ☐ somewhat serious ☐ moderately serious ☐ very serious

1. Having normal blood sugar levels before becoming pregnant would improve your chances of having a healthy baby:

☐ not at all ☐ a little ☐ somewhat ☐ a moderate amount ☐ a lot

*These questions ask about your beliefs about birth control and pregnancy prevention.*

1. Using birth control would prevent an unplanned pregnancy:

☐ not at all ☐ a little ☐ somewhat ☐ a moderate amount ☐ a lot

1. In the future, when I have sex, I intend to always use some type of birth control to prevent an unplanned pregnancy.

☐ definitely no ☐ probably no ☐ maybe ☐ probably yes ☐ definitely yes

*These questions ask about your confidence that you could do the thing named in each question.*

*“How confident am I that I could …”*

1. Get preconception counseling before I get pregnant?

☐ very unconfident ☐ unconfident ☐ maybe ☐ confident ☐ very confident

1. Change my insulin and diet to keep my blood sugar levels in normal range if I am planning a pregnancy (not yet pregnant)?

☐ very unconfident ☐ unconfident ☐ maybe ☐ confident ☐ very confident

1. Wait on becoming pregnant until my blood sugar levels are within the normal range?

☐ very unconfident ☐ unconfident ☐ maybe ☐ confident ☐ very confident

1. Convince my sexual partner that it is necessary to use birth control, even if he doesn’t want to use it?

☐ very unconfident ☐ unconfident ☐ maybe ☐ confident ☐ very confident

1. Use birth control each time I have sex when preventing a pregnancy?

☐ very unconfident ☐ unconfident ☐ maybe ☐ confident ☐ very confident

1. Delay sex if birth control is not available?

☐ very unconfident ☐ unconfident ☐ maybe ☐ confident ☐ very confident

*These questions ask about your experience today discussing reproductive health with the provider. Please be honest. Any feedback that you can give will be very helpful for us.*

1. What did you like about the book and/or the discussion?

1. What did you not like about the book and/or the discussion?

1. Did you like the colors used in the book? ☐ Yes ☐ No; please explain:
2. Did you like the diagrams used in the book? ☐ Yes ☐ No; please explain:
3. How was the length of the book? ☐ Too long ☐ Too short ☐ The length was right
4. How was the length of the discussion? ☐ Too much time ☐ Too little time ☐ The time spent was right
5. Would you prefer to read the book on your own and then be allowed to ask questions later? ☐ Yes ☐ No
6. Would you prefer to receive written materials that you can take home with you? ☐ Yes ☐ No
7. Would you prefer to use or receive written materials in another language? ☐ Yes ☐ No

*If yes, please specify which language(s)*: ______________________________________

1. Was there anything that made you feel uncomfortable, embarrassed, or upset in the book or during the discussion?

1. Is there anything else that could have been done differently to make the book or the discussion better?

**We are interested in learning about what you know about diabetes, puberty, pregnancy, and birth control now that you have talked with a provider. Make sure to read the questions carefully, and answer them as best you can. It is okay to get the answer wrong. Ask if you have a question on any item.**

☐ True ☐ False 1. Once a girl has begun having periods, she could become pregnant.

☐ True ☐ False 2. High blood sugar levels can cause irregular menstrual periods.

☐ True ☐ False 3. A girl/woman can become pregnant during her period.

☐ True ☐ False 4. A girl/woman cannot become pregnant the first time she has sexual intercourse.

☐ True ☐ False 5. To prevent pregnancy, birth control must be used each time a girl/woman has sex.

☐ True ☐ False 6. A girl/woman is most likely to become pregnant if she has sex about two weeks after her period.

☐ True ☐ False 7. A girl/woman with diabetes does not have an increased risk of vaginal infections.

☐ True ☐ False 8. A girl/woman with diabetes cannot have a healthy baby.

☐ True ☐ False 9. The target range for blood sugar during pregnancy in a girl/woman with diabetes is 100-200 mg/dl.

☐ True ☐ False 10. During pregnancy, the level of blood sugar in the fetus will be similar to the mother’s sugar.

☐ True ☐ False 11. High blood sugar levels during the first two months of pregnancy increase the risk of problems for the mom, but not the fetus.

☐ True ☐ False 12. If a girl/woman with diabetes has high blood sugar at conception (the moment they get pregnant), she has an increased risk of having a baby with birth defects.

☐ True ☐ False 13. A girl/woman with diabetes who has high blood sugar during pregnancy does not have an increased risk of miscarriage (sudden death of a fetus during pregnancy).

☐ True ☐ False 14. If a girl/woman with diabetes has high blood sugar during pregnancy, her baby could be a large baby, making delivery more difficult.

☐ True ☐ False 15. After a positive pregnancy test in a girl/woman with diabetes, the best way to prevent problems or harm to the fetus is getting blood sugar levels to the target range as soon as possible.

☐ True ☐ False 16. A girl/woman with diabetes has very few choices of birth control.

☐ True ☐ False 17. All birth control methods are less effective in girls/women with diabetes.

☐ True ☐ False 18. A girl/woman with diabetes cannot use any type of birth control pills.

☐ True ☐ False 19. A girl/woman with diabetes can use long-term birth control methods, such as an intrauterine device.

☐ True ☐ False 20. The sexual partner of a girl/woman with diabetes can use condoms.

☐ True ☐ False 21. Sex is like exercise and can cause low blood sugar (hypoglycemia) reactions.
