# Supplementary material for: Identifying and addressing gaps in reproductive health education for adolescent girls with type 1 diabetes
Source: PLoS One. 2018 Nov 6;13(11):e0206102. doi: 10.1371/journal.pone.0206102 (PMC6219771; doi:10.1371/journal.pone.0206102)
Supplement: S1 File — Included are the surveys utilized for the cross-sectional study of adolescents and health care providers, as well as the pre- and post-intervention surveys for the READY-Girls RHE study. (ZIP) [file pone.0206102.s001.zip › PLoS survey attach/Waiting room survey_PLoS.docx]

**We want to learn more about what you know about diabetes and reproductive health, so we can provide better care for you and other girls with diabetes. Please answer honestly -- your doctor and your parents will not see your answers. There are no right or wrong answers. You may leave any question blank if you do not want to answer. Completion of this survey implies that your parent/guardian has consented for you to participate in this research.**


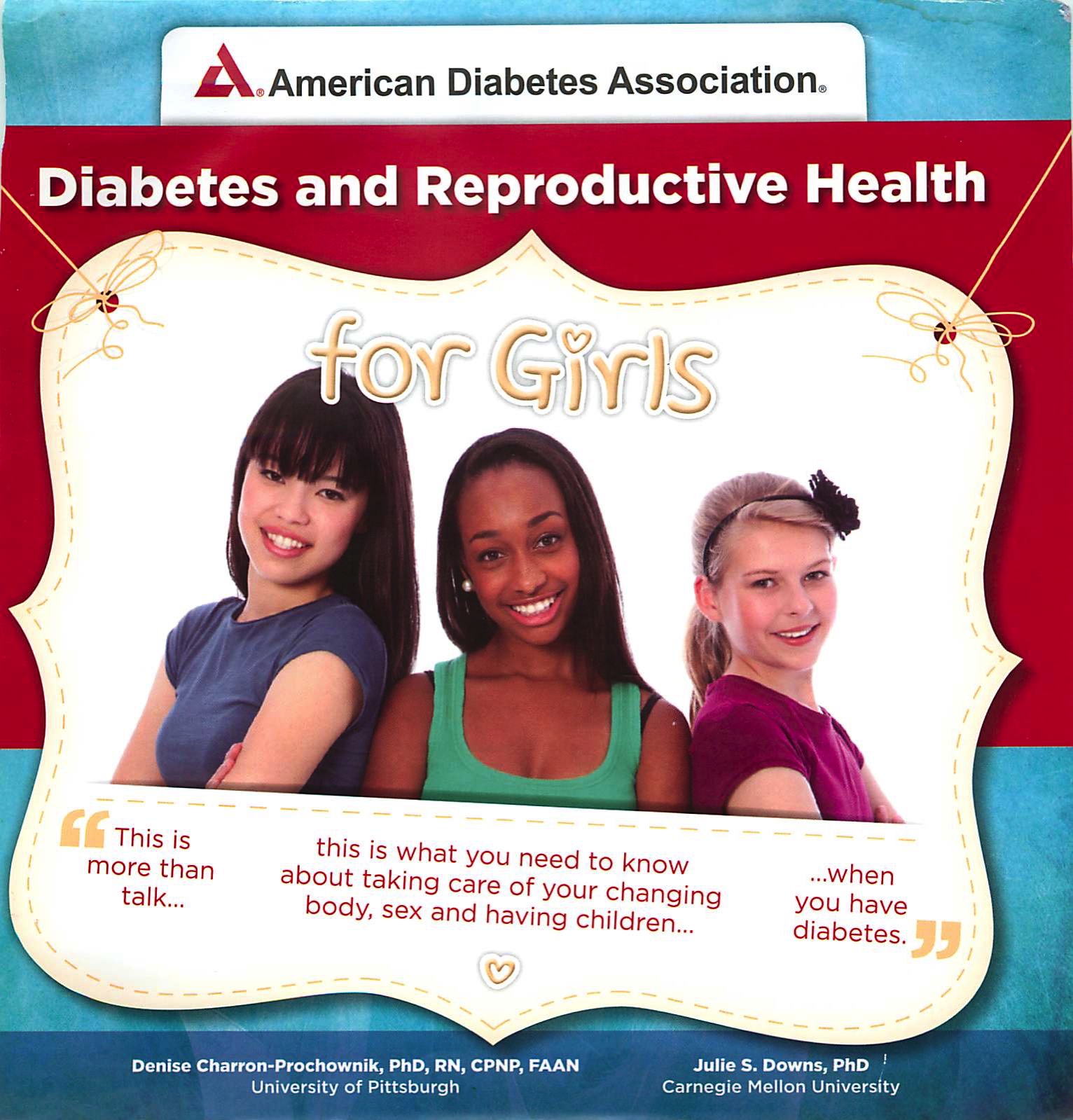

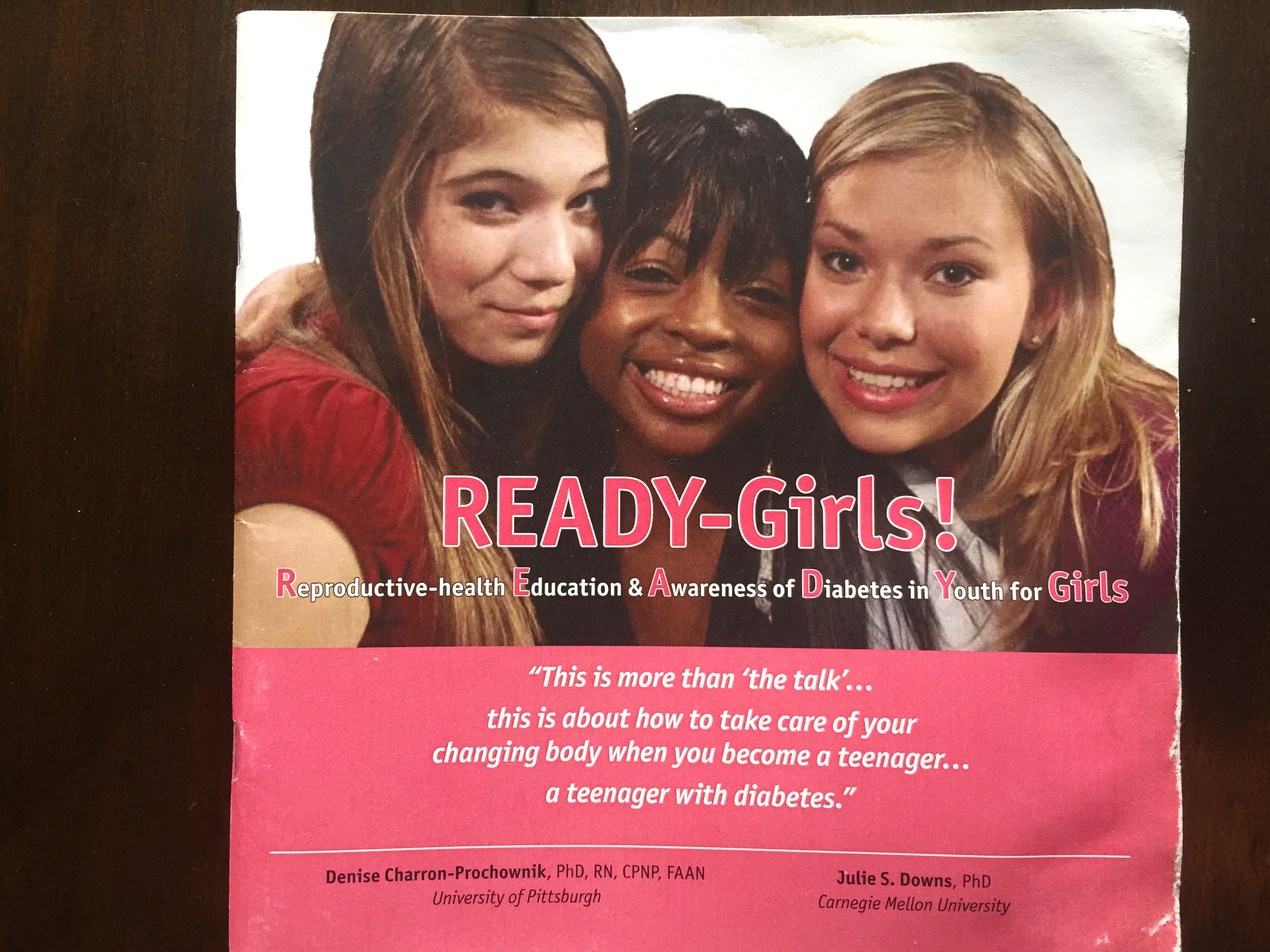


1. Have you received the READY-Girls information booklet? ☐ Yes ☐ No

***If the answer is yes, please stop here.***

1. How old are you? _______
2. How many years or months have you had diabetes? _______
3. What is your race/ethnicity?

☐ Caucasian (non-Hispanic) ☐ Hispanic / Latino ☐ Other:

☐ Black / African-American (non-Hispanic) ☐ Asian/Pacific Islander

1. Have you ever talked about how **diabetes affects puberty** with any of the following? (mark all that apply)

☐ diabetes doctor or nurse; if so, how old were you? _______

☐ diabetes educator; if so, how old were you? _______

☐ primary or regular doctor; if so, how old were you? _______

☐ obstetrician-gynecologist (Ob-gyn); if so, how old were you? _______

1. Have you ever talked about how **diabetes affects pregnancy** with any of the following? (mark all that apply)

☐ diabetes doctor or nurse; if so, how old were you? _______

☐ diabetes educator; if so, how old were you? _______

☐ primary or regular doctor; if so, how old were you? _______

☐ obstetrician-gynecologist (Ob-gyn); if so, how old were you? _______

1. Have you ever talked about **diabetes and birth control** with any of the following? (mark all that apply)

☐ diabetes doctor or nurse; if so, how old were you? _______

☐ diabetes educator; if so, how old were you? _______

☐ primary or regular doctor; if so, how old were you? _______

☐ obstetrician-gynecologist (Ob-gyn); if so, how old were you? _______

1. Have you ever talked about **diabetes and pre-conception counseling** (*medical care & advice given by a doctor, nurse, or educator before someone becomes pregnant / is planning a pregnancy*) with any of the following? (mark all that apply)

☐ diabetes doctor or nurse; if so, how old were you? _______

☐ diabetes educator; if so, how old were you? _______

☐ primary or regular doctor; if so, how old were you? _______

☐ obstetrician-gynecologist (Ob-gyn); if so, how old were you? _______

1. Do you currently use any birth control (to prevent pregnancy or for other reasons)? Please mark **all** that apply.

☐ Birth control pills ☐ Withdrawal (“pulling out”) ☐ Patch ☐ Rhythm/calendar method

☐ Condom (male) ☐ Diaphragm/cervical cap ☐ Nuvaring ☐ Arm implant

☐ Condom (female) ☐ Injections/shots ☐ Intrauterine device (IUD)

1. Have you voluntarily had vaginal sex with someone of the opposite gender?

☐ no ☐ yes; If yes, how old were you when you first had voluntary vaginal sex?

1. Are you currently, or have you ever been, pregnant?

☐ no ☐ yes; If yes, how many times have you been pregnant?

1. **Did anyone help you with this survey or look at your answers (a parent or someone else)?**

☐ no ☐ yes

**Thank you very much for taking this survey. Please put in the envelope and give it to the medical assistant when she takes your vitals.**
