# Supplementary material for: Identifying and addressing gaps in reproductive health education for adolescent girls with type 1 diabetes
Source: PLoS One. 2018 Nov 6;13(11):e0206102. doi: 10.1371/journal.pone.0206102 (PMC6219771; doi:10.1371/journal.pone.0206102)
Supplement: S1 File — Included are the surveys utilized for the cross-sectional study of adolescents and health care providers, as well as the pre- and post-intervention surveys for the READY-Girls RHE study. (ZIP) [file pone.0206102.s001.zip › PLoS survey attach/Parent, post-questionnaire_PLoS.docx]

**Now that you have talked with a diabetes provider about puberty, pregnancy, and birth control, we would like to know more about your attitudes and opinions, so we can provide better health care for your adolescent and other girls with diabetes. Please answer the questions honestly. There are no right or wrong answers. Ask if you have a question on any item.**

*These questions ask about your knowledge and beliefs about pre-conception counseling.*

1. What is preconception counseling and why is it important?

1. BENEFIT Receiving preconception planning (special medical care and advice) when planning a pregnancy would improve your adolescent’s chances of having a healthy baby:

☐ not at all ☐ a little ☐ somewhat ☐ a moderate amount ☐ a lot

1. BARRIER How difficult would it be for your adolescent to follow the preconception counseling advice given by a health professional (e.g. keeping blood sugar in the normal range, taking more insulin injections, etc.)?

☐ no problem at all ☐ a little ☐ somewhat ☐ a moderate problem ☐ a big problem

1. Which part of the preconception counseling would be the least difficult (easiest) for your adolescent to follow?

1. Which part of the preconception counseling would be the most difficult for your adolescent to follow?

*These questions ask about your beliefs about a possible pregnancy for your adolescent. In these questions, the word adolescent is used, but this does not assume that she will become pregnant as a teenager. These questions can also apply to any future pregnancy when your adolescent has become an adult woman.*

1. How much do you worry that your adolescent could become pregnant?

☐ not at all ☐ a little ☐ somewhat ☐ a moderate amount ☐ a lot

1. If your adolescent had an unplanned pregnancy, do you think that this problem would be

☐ not serious at all ☐ a little serious ☐ somewhat serious ☐ moderately serious ☐ very serious

1. If your adolescent developed health problems during a pregnancy, do you think that those problems would be

☐ not serious at all ☐ a little serious ☐ somewhat serious ☐ moderately serious ☐ very serious

1. How much do you worry that your adolescent could develop health problems during pregnancy?

☐ not at all ☐ a little ☐ somewhat ☐ a moderate amount ☐ a lot

1. If your adolescent’s baby developed health problems during a pregnancy, do you think that the problems would be

☐ not serious at all ☐ a little serious ☐ somewhat serious ☐ moderately serious ☐ very serious

1. How much do you worry that your adolescent’s baby could develop health problems during a pregnancy?

☐ not at all ☐ a little ☐ somewhat ☐ a moderate amount ☐ a lot

*These questions ask about your experience today discussing reproductive health with the provider. Please be honest. Any feedback that you can give will be very helpful for us.*

1. What did you like about the book and/or the discussion?

1. What did you not like about the book and/or the discussion?

1. Did you like the colors used in the book? ☐ Yes ☐ No; please explain:
2. Did you like the diagrams used in the book? ☐ Yes ☐ No; please explain:
3. How was the length of the book? ☐ Too long ☐ Too short ☐ The length was right
4. How was the length of the discussion? ☐ Too much time ☐ Too little time ☐ The time spent was right
5. Would you prefer to receive written materials that you can take home with you? ☐ Yes ☐ No
6. Would you prefer to use or receive written materials in another language? ☐ Yes ☐ No

*If yes, please specify which language(s)*: ______________________________________

1. Was there anything that made you feel uncomfortable, embarrassed, or upset in the book or during the discussion?

1. Is there anything else that could have been done differently to make the book or the discussion better?

1. Are there other things that your adolescent’s diabetes provider could do to help you and your adolescent feel better informed about puberty, pregnancy, and birth control related to diabetes?
